# The Cycle of EBV Infection Explains Persistence, the Sizes of the Infected Cell Populations and Which Come under CTL Regulation

**DOI:** 10.1371/journal.ppat.1003685

**Published:** 2013-10-17

**Authors:** Jared B. Hawkins, Edgar Delgado-Eckert, David A. Thorley-Lawson, Michael Shapiro

**Affiliations:** 1 Department of Integrative Physiology and Pathobiology, Tufts University School of Medicine, Boston, Massachusetts, United States of America; 2 University Children's Hospital (UKBB), University of Basel, Basel, Switzerland; 3 Dept. of Mathematics, Tufts University, Medford, Massachusetts, United States of America; Emory University, United States of America

## Abstract

Previous analysis of Epstein-Barr virus (EBV) persistent infection has involved biological and immunological studies to identify and quantify infected cell populations and the immune response to them. This led to a biological model whereby EBV infects and activates naive B-cells, which then transit through the germinal center to become resting memory B-cells where the virus resides quiescently. Occasionally the virus reactivates from these memory cells to produce infectious virions. Some of this virus infects new naive B-cells, completing a cycle of infection. What has been lacking is an understanding of the dynamic interactions between these components and how their regulation by the immune response produces the observed pattern of viral persistence. We have recently provided a mathematical analysis of a pathogen which, like EBV, has a cycle of infected stages. In this paper we have developed biologically credible values for all of the parameters governing this model and show that with these values, it successfully recapitulates persistent EBV infection with remarkable accuracy. This includes correctly predicting the observed patterns of cytotoxic T-cell regulation (which and by how much each infected population is regulated by the immune response) and the size of the infected germinal center and memory populations. Furthermore, we find that viral quiescence in the memory compartment dictates the pattern of regulation but is not required for persistence; it is the cycle of infection that explains persistence and provides the stability that allows EBV to persist at extremely low levels. This shifts the focus away from a single infected stage, the memory B-cell, to the whole cycle of infection. We conclude that the mathematical description of the biological model of EBV persistence provides a sound basis for quantitative analysis of viral persistence and provides testable predictions about the nature of EBV-associated diseases and how to curb or prevent them.

## Author Summary

Epstein-Barr virus (EBV) is a herpesvirus that establishes a lifelong persistent infection in virtually all human beings. This infection is a risk factor for the subsequent development of certain tumors and possibly also autoimmune diseases. In order to understand the origin of these diseases, it is necessary to first understand how EBV maintains persistent infection. We have used mathematical analysis to study this question. We find that the characteristic cycle of infected stages that EBV establishes in vivo allows it to persist stably at extremely low levels. This represents a consistent mathematical description of EBV infection and allows us to describe what must change to convert benign infection into pathogenic infection, as well as what kind of efficacy drugs and vaccines must have in order to be useful.

## Introduction

Epstein-Barr virus (EBV) is a herpesvirus that benignly infects more than 95% of the world's adult human population [1], but is occasionally associated with certain tumors including 3 forms of lymphoma [2]. One prominent feature of EBV is that it persists as a lifelong low-level infection in the memory B-cells of healthy carriers [3], [4]. Our laboratory has measured the level of infection in the peripheral blood memory B-cells of healthy carriers over the course of decades ([5], [6] and unpublished observations) and shown that it remains stable. If there is a real decline (or expansion), it is happening too slowly to detect. Persistent infection is also associated with an active humoral and cellular immune response by the host that is also stable over time [1], [7]. We see this stability as a balance between infection and the immune response which returns to equilibrium when perturbed. Two biological models have been proposed to account for this persistence: the germinal center (GC) model [4], [8] and the direct infection model [9], [10].

**Figure 1:**
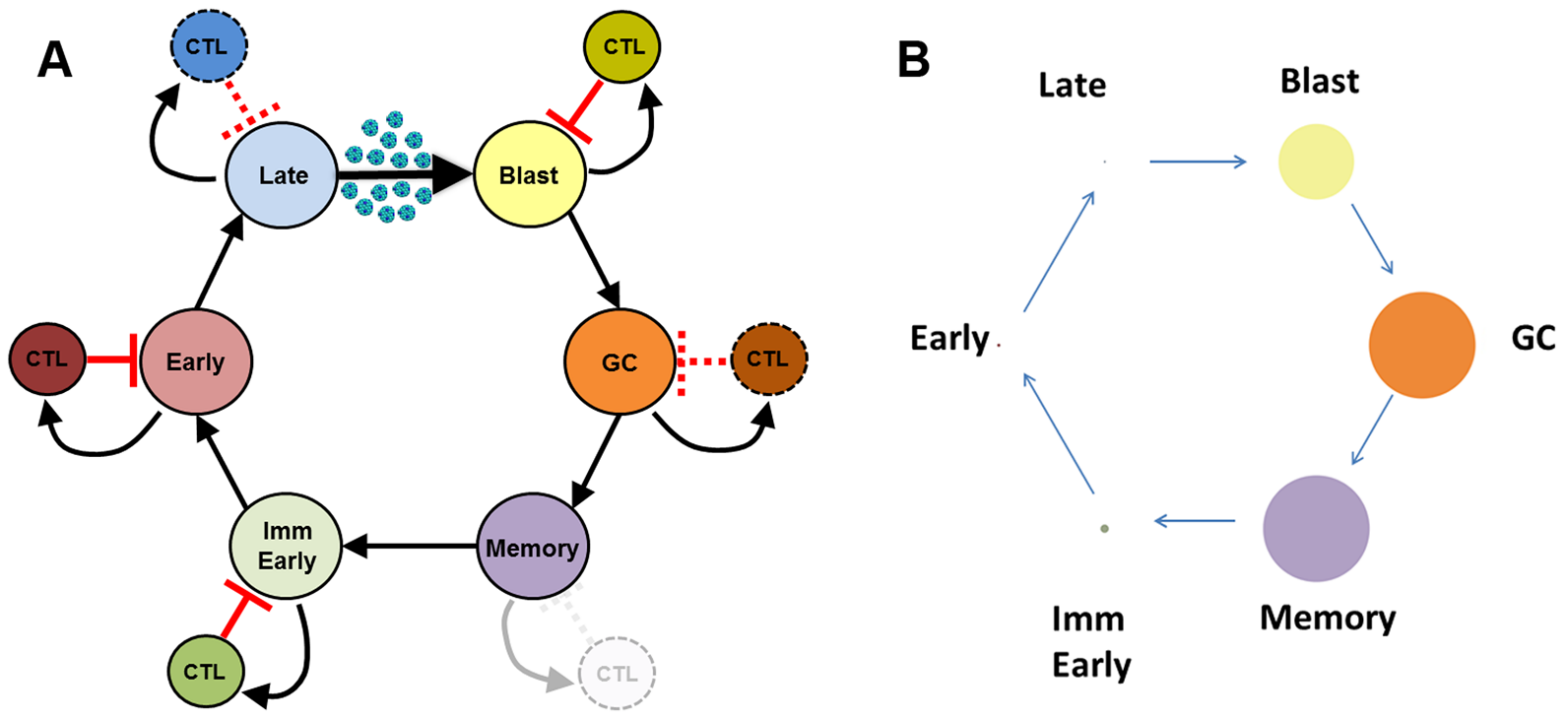
EBV biological model. A) Newly infected B-cell Blasts move into the follicle and enter the GC, where they continue to divide as EBV-infected GC B-cells before exiting into the periphery as latently infected memory B-cells. A small subset of these are induced to undergo lytic reactivation, progressing through the lytic stages Immediate early, Early and Late before finally bursting and releasing infectious virus that may be amplified through infection of the epithelium (not detailed in the model) but ultimately culminate in the infection of new naive B-cells which become Blasts, thus completing the cycle. Theoretically each stage has the capacity to generate a CTL response. The Blast, Immediate early and Early stages are always regulated, while the GC and Late stages may not always be regulated [7], [32], [33]. Memory is never regulated under normal biological conditions [4], [8], [11]. This model of EBV biology is used to generate the CPM framework presented in this paper. B) Infected populations as displayed as circles whose area is proportional to their frequency within all tonsils (1∶5∶1.5×10^2^∶10^4^∶10^4^∶0.5×10^4^, Late∶Early∶ImmEarly∶Memory∶GC∶Blast). This graphic highlights the very large range in the sizes of these populations.

The GC model proposes that EBV persists by exploiting normal B-cell biology. This involves new latently infected B cells passing through a series of differentiation stages, each employing a discrete viral gene transcription program (Figure 1). Thus, EBV directly infects naive B-cells, causing their activation into proliferating latently infected Blasts. At this stage the virus expresses all nine known latent proteins, a condition referred to as latency 3 or the growth transcription program. These cells then move into the germinal center (GC) to participate in the GC reaction. Here they express a more restricted pattern of latent proteins referred to as latency 2 or the default program. Eventually these cells leave as latently infected memory B-cells that either only express the viral genome tethering protein EBNA1 (known as the EBNA1 only program or latency 1) or no viral proteins at all. The later state is referred to as latency 0 or the latency program. The memory compartment has been considered the site of long-term persistence because the virus is quiescent [11], and therefore invisible to the immune response. At any time a small subset of latently infected memory B-cells initiates lytic reactivation in association with terminal differentiation signals [12], [13]. Reactivation of the virus is subdivided into three discrete phases; Immediate early when the transcription factors initiating viral replication are expressed, Early when the proteins involved in viral DNA replication are produced, and Late when viral DNA and structural proteins are assembled into virions [14]. Ultimately this leads to the release of infectious virus that can either be shed or infect new naive B-cells, thus completing the cycle. Each stage of this cycle has been demonstrated experimentally [13], [15], [16] and, with the exception of the memory compartment, is potentially regulated by the immune response [7]. Thus, the GC model accounts for all the latent and lytic stages of the virus and thereby provides an explanation for the origin of EBV-associated lymphomas. For example, Burkitt's lymphoma and Hodgkin's disease are believed to descend from latently infected GC B-cells which have failed to successfully differentiate into a resting memory state (for a detailed discussion of this issue see [2]).

The second model, proposed by Rajewsky and coworkers [9], [10], holds that EBV directly infects memory B-cells. Although proposed over 10 years ago, no evidence has subsequently been evinced to explain the mechanism behind this model. Unlike the GC model it does not account for the four well-defined transcription programs/states of latent EBV infection, intermediate states between newly infected and persistently infected memory B-cells have not been identified in vivo, the model does not account for the origin of EBV-infected tumors and the basis for viral reactivation remains unexplained. Furthermore, predictions of the direct infection model were incorrect when tested experimentally and instead supported the GC model. For example, infected GC B-cells express the viral default transcription program (latency 2) in vivo [16], [17] (as predicted by the GC model), not latency 3 (as predicted by the direct infection model [18]), and in a transgenic mouse model one of the EBV latent proteins expressed in the GC (LMP2a) was able to drive B-cells to form GCs in the absence of antigen [19]. Thus, the direct infection model remains ill-defined and unverified at the biological level, and therefore difficult to test mathematically.

Like most biological models the GC model is descriptive and, as such, is not quantitative. However, unlike the direct infection model it is sufficiently detailed for mathematical testing and analysis [20], [21], [22], [23], [24], [25], [26]. Mathematical approaches to studying host-pathogen interactions have increased steadily in the last four decades. (For entry into the corresponding body of literature, we recommend [27], [28], [29], [30], [31]). Mathematical analysis can be rigorous (i.e., comprehensive, thorough and exact) and a good model should be able to withstand such analysis. Thus, if a biological model is not mathematically consistent, i.e., not capable of being described mathematically, it cannot be correct. Therefore, one test of the validity of a biological model is to see if it is mathematically sound. If such a mathematical description can be established, it provides a powerful quantitative tool for analyzing the infection process. For the first time this would allow us to quantitatively define the biological constraints on the behavior of the virus, determine just how stable the persistent infection is, and where and how aggressively interventions must be applied to alleviate and/or prevent infection and disease. We have provided such a detailed mathematical description of the GC model [21]. The current paper depends critically on this work where we modeled the interactions between a host and a pathogen, such as EBV, which transits a cycle of antigenically distinct stages. We refer to this model as the cyclic pathogen model (CPM). The paper describing CPM is highly technical and may not be accessible to many working biologists, therefore a review of the relevant material is presented in Boxes 1, 2 and 3.

**Box 1. The Model**

For each infected stage, we model the following processes: the rate at which it is lost to become (or produce) the next stage; the gain in this production (e.g., the loss of one lytic cell may produce many infected blasts); the net birth or death rate of the stage; the net antigenicity of the stage, i.e., its efficacy in inducing CTL activation and proliferation; and the killing efficiency of each stages' cognate CTL population. In addition, we assume that in the absence of antigen, CTL populations decline at a rate that is common across all stages. Each of these processes appears in the equations as a rate governing change in either the population of infected cells or the CTLs that recognize them. These parameters and the equations are given in Box 2.

When a pathogen is introduced into a naïve host, it makes copies of itself. We use *R*_0_ to denote the average number of fresh copies produced by each introduced copy. Clearly, to establish infection, a pathogen must have *R*_0_>1.

We are interested in the fixed points of the system when the pathogen is able to establish a stable persistent infection. As a mathematical system, the CPM has 2*^n^* fixed points where *n* is the number of infected stages, so the six-stage model of EBV has 2^6^ = 64 mathematical fixed points. Many of these are non-biological; that is, they require a negative number of CTLs. Given the long-term stability of EBV infection, we are interested in stable equilibriums of the system.

What makes an equilibrium stable or unstable? To say that a state is in equilibrium means that if a system is not subject to disturbance, it will stay in that state. To say that an equilibrium is stable means that if the system is disturbed from that state, it will return to that state whereas when an unstable equilibrium is perturbed, it does not return.

The perhaps surprising result of the CPM analysis is that for any set of parameter values, i.e., controlling rates, the system has exactly one stable equilibrium. The mathematics of this equilibrium are described in Box 3. This equilibrium is determined by four basic observations:

- If a stage comes under immune regulation, its set point at equilibrium will be exactly the population size necessary to provoke a sufficient CTL response to control the stage. At this point CTL proliferation will exactly balance CTL attrition.
- If a stage is not under immune regulation, its population size is determined by the rate at which it is produced from the previous stage and its average lifespan before differentiating or dying. We call this its follow-on population.
- If a stage is unregulated and its follow-on population is less than it would be if it were regulated, it is insufficient to support regulation by the immune response. Put another way, the immune response at that stage is starved of sufficient antigen and a CTL response cannot be sustained.
- If a stage is unregulated and its follow-on population is greater than it would be if it were regulated, the system is in an unstable state; it is like an ecosystem that is susceptible to invasion by a new species. If a response to this stage is introduced, it will expand and drive this stage down to its regulated population size, thus shifting the state of the entire system.
- The system achieves stability only once all CTL responses that can be activated have been activated.

This characterization of the stable fixed point allows an efficient computation which, when given a set of values for the parameters, determines the stable fixed point. This gives an effective way of testing our model and the values we have estimated as biologically tenable. For each set of values, we can compute the stable fixed point and pattern of regulation.

**Box 2. The Mathematics of the Model**

We assume that the pathogen transits *n* distinct stages. We then have 2*n* populations, the infected stages, *S*_1_,…,*S_n_* and cognate CTL populations *T*_1_,…,*T_n_*. These interact via the 2*n* equations, one pair for each value *i* = 1,…,*n*. These are taken cyclically so that the stage “previous” to stage *i* = 1 is stage *i* = *n*. Thus, for the purposes of numbering these equations, we treat “1-1” as *n*.
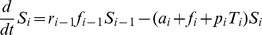


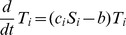
Here for each *i*,

- *f_i_* is the rate at which stage *i* is lost to become (or produce) stage *i*+1.
- *r_i_* is the gain, and is equal to 1 except for the late lytic stage.
- *a_i_* is the net death rate. If the stage proliferates, this number is negative.
- *p_i_* is the killing efficiency for the CTLs at stage *i* and encapsulates the likelihood of a CTL finding its target, forming a stable conjugate, and the efficiency of killing.
- *c_i_* is the net antigenicity of stage *i*, that is, the efficiency with which stage *i* maintains CTL activation and provokes CTL proliferation.
- *b* is the rate of decay of the CTL response in the absence of antigen.

**Box 3. The Mathematics of Equilibrium**

- At equilibrium we have
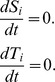

- A stage *S_i_* is *regulated* if *T_i_*≠0. If a stage is regulated, its population is determined by its net antigenicity and the decay rate of the T-cell response
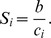
Since *b* is common across all stages, the relative sizes of the regulated stages depends solely on their net antigenicities (see also [44]).
- If a stage is unregulated, its population is determined by the size of the previous stage and its *follow-on* constant. This gives the *follow-on population* which is the product of its rate of production times its average lifespan,
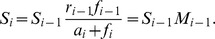

- If the follow-on population is less than 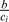, it is insufficient to support immune regulation. The immune response is *starved* of sufficient stimulation (see also [44]).
- If an unregulated population is larger than 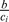, this is unstable. Introduction of a single CTL directed against this stage will proliferate producing a new equilibrium in which this stage is regulated and its population has been reduced to 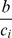.
- It may happen that whenever stage *S_i_* is regulated, the follow-on population at stage *S_j_* is insufficient to support regulation. In this case we say that *S_i_ starves S_j_*.
- The pattern of regulation of the stable equilibrium is determined as follows:

 

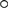
 if a stage is not starved by any other stage, it will be  regulated.

 

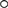
 if a stage is starved by some other stage, it will not be  regulated.

The most interesting conclusion of CPM was that while for any given set of parameter values there are many potential equilibrium states, only one has non-negative populations (and is therefore biologically meaningful) and is stable. We propose here that this unique, biologically meaningful, stable fixed point corresponds to long-term persistent infection by EBV. If correct, then the essential features of a cyclic host-pathogen interaction like EBV's can be accurately represented mathematically, which in turn shows that the biological model is mathematically consistent.

In our application of the CPM to the study of EBV, we assume that persistent infection has reached a stable equilibrium. We also assume that all the infected stages in the cycle possess some level of antigenicity. Whether an immune response arises to a particular infected stage depends in part on the number of cells at that stage. If it is too high, the immune response will drive the number down to the point where it is just sufficient to sustain the response. Conversely, if the number is too low, the cells will fail to establish or sustain an immune response. In this event there are two potential outcomes. In one instance, the infected population rises until it generates and is counterbalanced by an immune response. In this case the stage is regulated by the immune response. Alternatively, if the population is limited, for example by its rate of production from the previous stage(s), it may already be at a steady state level and therefore will not sustain an immune response. In this case the population is not regulated by the immune response but by the rate of follow-on from the previous stage. For any given set of parameters, we use the term “pattern of regulation” as the final outcome of which stages are regulated by the immune response (regulated stages) and which are regulated as follow-on populations (unregulated stages) for that particular set of parameters.

In this paper we have sought to test the hypothesis that the unique biologically meaningful stable fixed point predicted by the CPM corresponds for EBV to long-term persistent infection. To achieve this, we challenged the model with the full range of biologically plausible values for the model parameters and asked if the observed regulation patterns are biologically valid.

## Results

### The unique stable fixed point of the CPM describes persistent EBV infection

We wished to test the hypothesis that the unique stable fixed point predicted by the CPM is a description of EBV infection. To achieve this, we have estimated the range of values for all the parameters needed to compute this stable fixed point (for details see Methods and Supplementary Table S1). Given a value for each parameter, we can calculate the unique biologically meaningful stable fixed point determined by that particular set of parameters and ask if it is biologically valid. Specifically, we can determine the pattern of regulation, i.e., which stages fall under direct regulation by the immune response and which do not.

For EBV we have a six-stage model of infection (naive Blast, GC, memory, Immediate early lytic, Early lytic and Late lytic) where each stage may or may not be regulated by the immune response. Therefore, there are in theory 2^6^ = 64 possible combinations of regulated and unregulated stages. In actual EBV infection, the memory stage does not express CTL targets so presumably is never regulated [4], [8], [11]. Analysis of responses to latent and lytic antigens [7] reveals that CTL recognizing antigens expressed:

1. in the blast stage (latently infected naive B-cells expressing all latent proteins) are always detected [32].
2. in the GC (LMP1 and LMP2a) are only detected in a subset of individuals (∼60%) [32].
3. in the Immediate early and Early stages of virus production are always detected [33].
4. in the Late stages of virus production are only detected in a subset of individuals (∼28%) [33].

Therefore, of the 64 hypothetically possible patterns of regulation, only 4 are biologically credible. In order of prevalence they are:

1. Memory stage alone is not regulated - Blast, GC, Immediate early, Early and Late lytic are regulated.
2. Memory plus GC stages are not regulated - Blast, Immediate early, Early and Late lytic are regulated.
3. Memory plus Late lytic stages are not regulated - Blast, GC, Immediate early and Early are regulated.
4. Memory plus GC plus Late lytic are not regulated - Blast, Immediate early and Early are regulated.

**Figure 2:**
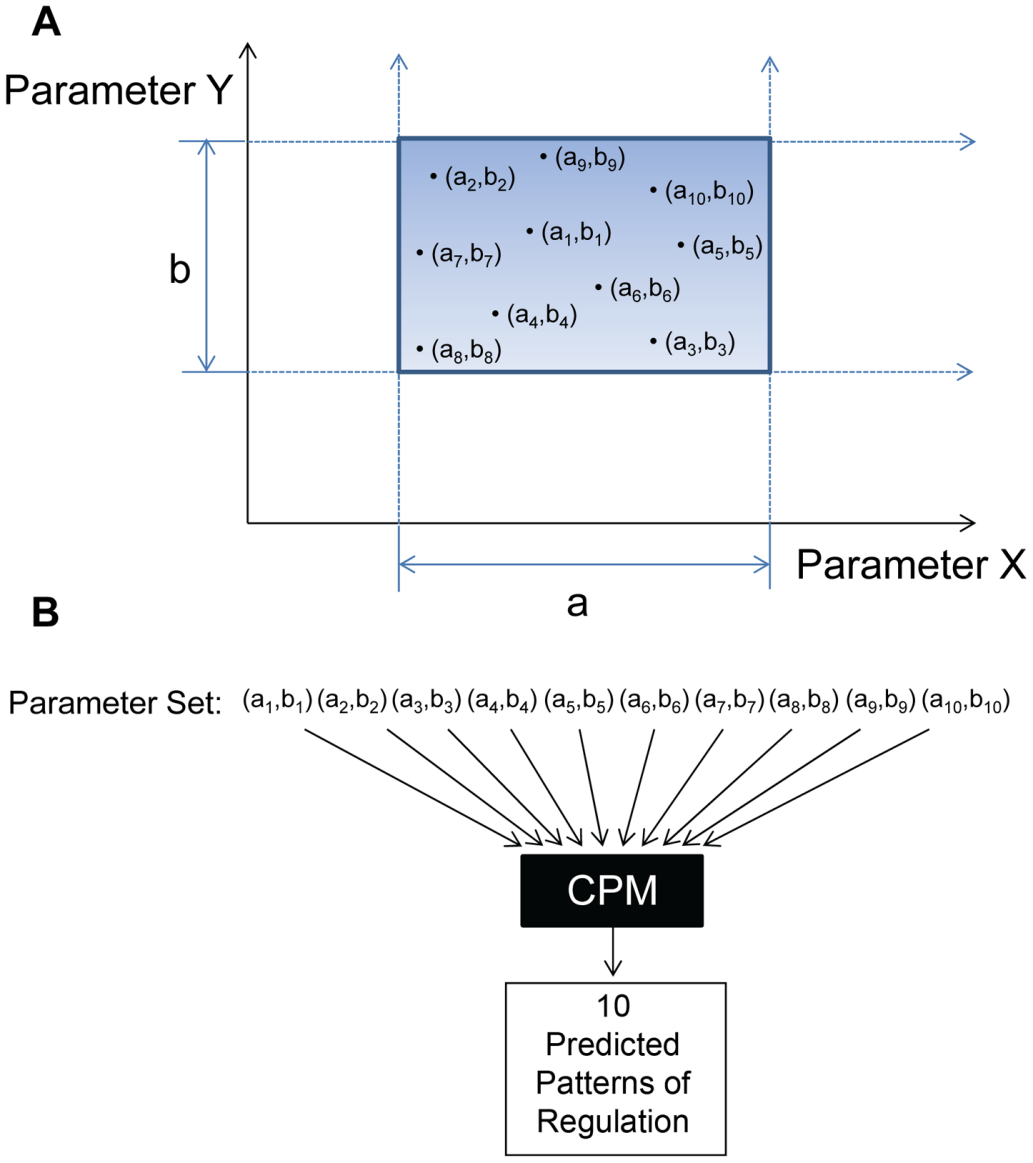
Diagrammatic representation of our test procedure for CPM. A) Here we describe the methodology for just 2 parameters, X and Y. a and b represent the range of biologically tenable values for these two parameters. From this we can project a 2-dimensional parameter space that consists only of all the biologically tenable combinations of the two parameters. We can then sample random points in this space (in this case 10). B) Each point is a parameter set which consists of a single value for each of the two parameters (parameter values). These can then be used to interrogate the model and predict outcomes. In our model there are actually 25 parameters generating a 25 dimensional parameter space (the parameter cube) from which we randomly sampled 10,000 sets of parameters.

**Figure 3:**
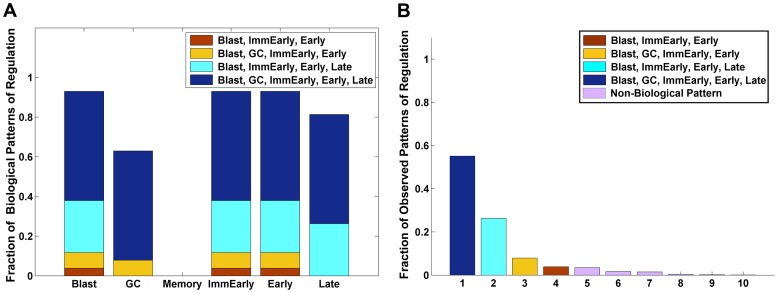
Biologically plausible immune patterns of regulation. Out of 10,000 randomly chosen parameter sets from the physiologically tenable parameter space, for this particular run, 93.1% produced biologically plausible patterns (described in the insert). A. Columns represent the total fraction of patterns for which each stage was regulated. B. The patterns of regulation ordered by frequency of occurrence. Note the four highest are the biologically plausible patterns and the highest is the most likely to occur biologically.

Our model contains 25 parameters that affect the size of the infected populations (for a detailed description and discussion see Methods and Supplementary Table S1). We have identified the biologically credible range of values for each of these parameters (see Methods). Together, the combined ranges for these parameters can be thought of as generating a 25 dimensional parameter space. This space consists of all the possible combinations for the plausible values for our 25 parameters; we refer to this as the “parameter cube”. We then tested the validity of our model by sampling 10,000 random points in that cube (i.e., 10,000 randomly chosen combinations of biologically tenable values for the 25 parameters) and computing the pattern of regulation at the stable fixed point for each set of parameters. A simplistic version of this approach for 2 instead of 25 parameters is shown in Figure 2. For a typical run of 10,000 randomly selected parameter sets, the four most prominent patterns of regulation we found are shown in Figure 3A and B. In order of prevalence they were:

1. Memory stage alone is not regulated – 55.1%.
2. Memory plus GC stages are not regulated – 26.3%.
3. Memory plus Late lytic stages are not regulated – 7.9%.
4. Memory plus GC plus Late lytic are not regulated – 3.8%.

Thus a full 55.1% gave the most common pattern of regulation seen biologically, i.e., where all stages are regulated by the immune response except memory. Furthermore, of the 64 possible patterns of regulation, the top four patterns were the 4 biologically credible ones, and they accounted for essentially all of the random sample of parameter sets (93.1%). Of the non-biological patterns, 6 accounted for the remaining 7% of predicted patterns and 54 patterns were never detected. We speculate that these biologically implausible patterns of regulation arise because there may be combinations of plausible values for the parameters that are not consistent with each other.

Direct comparison of model predictions with CTL studies are also informative. The model predicts that the Blast, Immediate early and Early stages are almost always regulated (>95% of the time), as is seen experimentally. Similarly the model predicts that the GC is regulated 63% of the time which is very close to the 60% predicted from CTL studies [32]. Taken together, these results provide strong quantitative validation of the model. The case for Late lytic is less convincing since the model predicts regulation 88% of the time, but CTL studies only report detection 28% of the time [33]. Thus the model is qualitatively accurate (the Late lytic stage is not always regulated), but either quantitatively imprecise in this area or the biological data are not accurate. For example, it has been suggested by the original authors that 28% is an underestimate (Hislop, personal communication). Clarification of this point experimentally will provide a test of the accuracy and predictive power of the model.

The model's prediction that the Late lytic stage is not always regulated makes an important point in terms of understanding how the model works. The model predicts that at equilibrium, the size of a population regulated by the immune response is inversely proportional to its net antigenicity. This is because the more antigenic a population, the fewer cells it takes to stimulate a controlling T-cell response. Based on the observed population sizes of Immediate early (∼50–500 cells), Early (∼5–50 cells) and Late (∼2–10 cells) lytic populations in all of the tonsils at equilibrium ([13] and Supplementary Table S1), i.e., during persistent infection, the model predicts that net antigenicity should increase across the lytic stages, with the Late lytic population being the strongest. Thus, it is noteworthy that published observations on the avidity of CTLs to these stages demonstrates that Late CTLs are indeed significantly more avid than Immediate early or Early [33]. Since the Late stage has a higher antigenicity than any other stage, a superficial analysis might predict that it should always be regulated by the immune response. However, the CPM model states that, no matter how antigenic a state is, if the size of the population is below the level necessary to trigger an immune response, it will only be regulated as a follow-on population, that is to say the rate at which it is produced from the previous stage combined with its average lifespan. The model predicts that in about 12% of cases there will be insufficient Late lytic cells to stimulate a detectable CTL response. This demonstrates that net antigenicity alone does not predetermine which stages in the model are regulated and which are not.

### The role in EBV persistence of viral quiescence in the memory compartment

**Figure 4:**
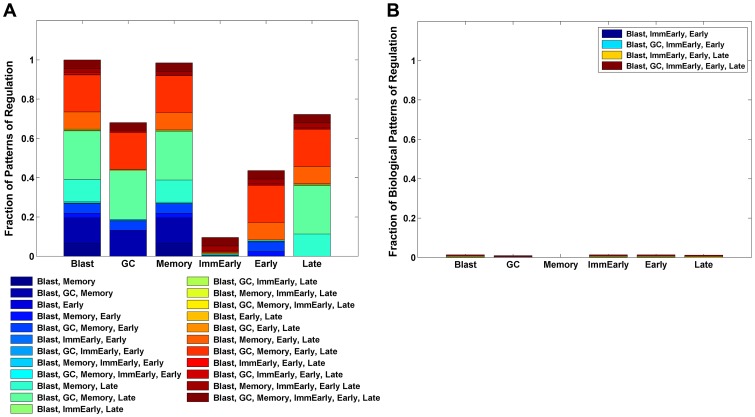
Testing the consequences of an immunologically invisible memory stage. A) The same analysis was performed as in Figure 3, with the exception that the memory compartment was allowed to be antigenic. The most frequent patterns of regulation seen are shown in the insert. B) The same analysis as A, but showing only the fraction where the four biologically plausible patterns of regulation were seen.

One central premise of the biological model is that EBV can persist because it resides quiescently in resting memory B-cells that cannot be recognized by the immune response. The CPM makes a different prediction, namely that even if the memory compartment was antigenic the virus could persist. However, the structure of persistence in terms of population numbers and responses would be very different from what is seen biologically. An example is shown in Figure 4. Here the same analysis was performed as in Figure 3, but with the memory compartment being assigned a much greater antigenicity. The picture that emerges is complex (Figure 4A) and dominated by non-biological patterns of regulation (Figure 4B). Perhaps the most striking outcome being that in ∼90% of the cases the Immediate early stage is no longer regulated despite being strongly antigenic. Cleary this is a non-biological result, since we know that biologically the Immediate early stage is always under regulation [7], emphasizing again the point that net antigenicity alone does not decide if a stage will be regulated by the immune response.

This result produces a shift in our understanding of persistence away from relying on the poorly antigenic nature of the memory compartment towards the importance of the whole cyclic nature of the infection. Expressed more generally, it is not the CTL response (or lack thereof) to certain, specific stages that explains EBV persistence. EBV could persist no matter the pattern of regulation. Rather, it is the CTL response (or lack thereof) to certain, specific stages that defines the way persistence looks.

### Biological insights - The memory compartment

**Figure 5:**
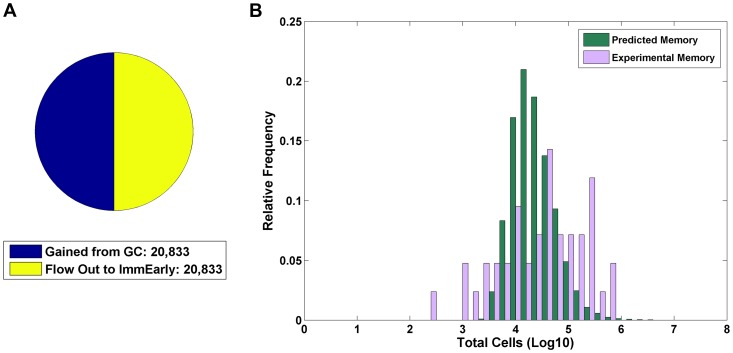
Analysis of the memory compartment. A) The size of the infected memory compartment and flow rates through this stage is shown graphically as a pie chart. The left half shows gains by the population and the right half shows the losses. Since the system is at equilibrium, the population size is constant and the gains must equal the losses, i.e., the size of the two halves of the pie chart must be the same. B) To test how well the range of values predicted for the memory compartment by CPM compared to what is seen biologically, we calculated the predicted steady state size of the memory compartment for 10,000 randomly chosen parameter sets (green bars) and compared this to the actual number of EBV infected memory B-cells in Waldeyer's ring for 42 independent tonsils from persistently infected individuals (purple bars). The predicted values have a similar mean and median value as compared to the experimental data (log mean: biological 4.48; CPM 4.36; log median: biological 4.65; CPM 4.31; p-value = 0.079 using a two-sided, unpaired, two-sample Mann-Whitney test).

The model produces detailed predictions about the sizes and flow rates through each stage for any given set of parameters (i.e., point in the parameter cube). A relatively simple way to demonstrate this is with pie charts, where the left half shows the flows into the population and the right half shows the flows out. Since the system is stable, i.e., at equilibrium, the population size for each stage is constant and the flows in must equal the flows out, i.e., the size of the two halves of the pie chart must be the same. Net gain can occur either from input from the previous stage or as the end product of cell division, i.e., proliferation. Net loss can occur via death, killing by CTLs, differentiation to the next stage or as loss to cell division (for convenience we consider cell division to be the consequence of loss of one cell and the appearance of two new cells). The simplest case is the memory compartment, which biologically is never regulated by the immune system. We have proposed previously that the infected memory compartment is regulated by normal memory B-cell homeostasis. In this case, we assume that death and proliferation are exactly balanced, producing the pie chart shown in Figure 5A where net input from the GC stage effectively equals net output to the Immediate early stage (see Methods for further discussion). We can also calculate the predicted population size and the flow rate through the stage (values shown in the figure come from a particular set of typical parameters). In our analysis the memory population is never regulated by the immune response, rather its level is predicted by CPM to be a complex outcome of all the interactions and rates throughout the model and, as such, an emergent property of the whole model, i.e., not pre-programmed or governed by specific model parameters. Currently, there is no way to measure the actual flow rate through any given stage, but we are able to measure the population sizes. To test how well the range of values predicted by CPM compared to what is seen biologically, we calculated the steady state size of the memory compartment for each of the 10,000 randomly chosen parameter sets. The result is plotted as a histogram in Figure 5B (green bars). Superimposed upon it is a histogram of the actual number of EBV infected memory B-cells in Waldeyer's ring for 42 independent tonsils from persistently infected individuals (purple bars). As may be seen, the full range of CPM predicted values falls within the actual range of biological measurements. Furthermore, the predicted values have a similar log mean and log median value (log mean: biological 4.48; CPM 4.36; log median: biological 4.65; CPM 4.31; p-value = 0.079 using a two-sided, unpaired, two-sample Mann-Whitney test). The major difference is that the biological values have a somewhat wider range (std. dev. of log values: biological 0.83; CPM 0.40). Since this distribution of values for the size of the memory population is derived only as an emergent property of the model, we conclude that the accuracy of the predicted range serves as good validation both for the theoretical underpinning of the CPM model and for the credibility of our parameter set.

### Biological insights - The GC compartment

**Figure 6:**
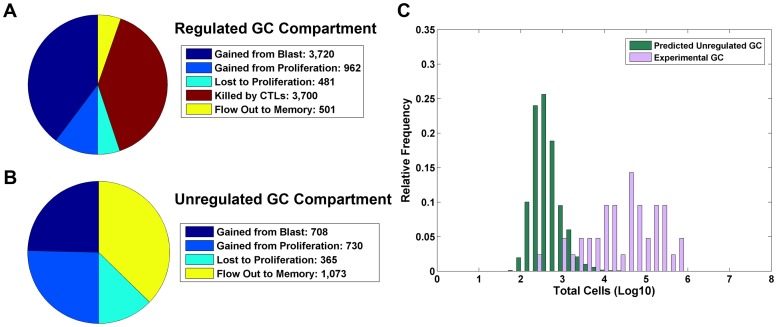
Analysis of the GC compartment. A). The size of the infected-GC compartment and flow rates through this stage in an example where the infected-GC population is regulated by CTLs is shown graphically as a pie chart. The left half shows gains by the population and the right half shows the losses. Since the system is at equilibrium, the population size for each stage is constant and the gains must equal the losses, i.e., the size of the two halves of the pie chart must be the same. Gain can occur either from input from the previous stage or as the end product of cell division, i.e., proliferation. Losses can occur via death, killing by CTLs, differentiation to the next stage or as input into cell division (it is simplest to consider cell division to be the consequence of loss of one cell and the appearance of two new cells). B) The same analysis as A, however for an example where the infected-GC population is not regulated by CTLs. C) The predicted size of the infected-GC population is shown for the cases in which it is not regulated by the immune response (3,193 out of 10,000 random trials; green bars). In purple, we have plotted the experimentally measured size of the infected-GC population from 42 independent tonsils from persistently infected individuals.

We can apply a similar analysis to the GC compartment. We have seen that CPM predicts, in good agreement with experimental data [32], that the GC compartment is regulated approximately 63% of the time. Pie charts for both regulated and unregulated conditions are given in Figure 6A and B respectively. These also reiterate the key features of the model. Thus, in Figure 6A (the regulated condition), the rate at which infected GC B-cells are produced from the previous stage and by proliferation is greater than the rate at which they are lost to the next stage. Thus, the size of this stage will increase until it is above the threshold for triggering an immune response. Above this threshold the immune response will expand and drive the infected population back down to the threshold level. So, at equilibrium a regulated population will be at the threshold level where there are just enough infected cells to drive the immune response and just a sufficient number of T-cells to limit the population.

The size of the regulated population is a model assumption because the level to which the immune response limits it is a direct function of its net antigenicity which is given by the model parameters *c_i_* and *b* in the equation 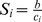. However, if the equilibrium population size is not sufficient to induce an immune response, i.e., below the threshold, then for this unregulated population the rate of production (from the previous stage and proliferation) must equal the rate of loss (to the next stage); this is the condition displayed in Figure 6B. As with the memory compartment, the size of this GC population is emergent, i.e., a complex outcome of all the interactions and rates throughout the model - not governed by model parameters. As such, it provides a vehicle to further test and validate the CPM. We can ask what the range of these values is for the ∼30% of our parameter sets that produce an unregulated GC population and compare it to the 42 independent measurements we have made on biological samples. Since we know that failure to be regulated by the immune response arises if a population does not achieve a large enough size, we would predict that the range of sizes for unregulated GC populations should reside at the lower end of the range of measured values. The result is shown in Figure 6C. The purple histogram shows the distribution of actual measured values and the green histogram shows the values predicted by CPM for the parameter sets where the GC compartment is unregulated by the immune response. As predicted, the values from CPM lie within those measured biologically, but at the lower end of the range. Once again this provides validation both for the model and the parameter set we have chosen to test it. Since most of the biologically measured values are larger than those predicted for unregulated GC populations, this model states that these must be regulated levels, i.e., in the majority of individuals the infected population of GC B-cells is regulated by the immune response.

### Biological insights - The Blast compartment

**Figure 7:**
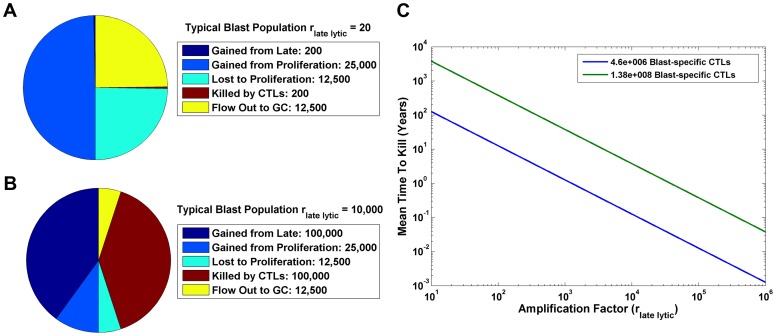
Analysis of the Blast compartment. A and B) Flow rates for the Blast stage are shown using the amplification factors of 20 (A) and 10,000 (B). C) The predicted mean time between killing for CTLs specific for Blasts in Waldeyer's ring is plotted against a wide range of amplification factors. The green line represents our high estimate for this CTL population, while the blue line is the low estimate.

The simplest model for reinfection is that each bursting B-cell releases infectious virions that infect adjacent B-cells. Since the avidity of EBV for its receptor on B-cells is so high, it is unlikely that the virus from each lytic cell would travel beyond the first layer of surrounding B-cells, i.e., infection of approximately 20 B-cells, to become Blasts. An example of the net gains and losses to the Blast stage based on this scenario is shown in Figure 7A. In this case, approximately 10 cells undergo lytic reactivation per day resulting in ∼200 freshly infected cells, a vanishingly small contribution compared to the approximately 12,500 daughter cells produced daily by proliferation. In order to balance the two sides (i.e., at steady state), it is necessary to invoke that approximately 200 Blasts are killed per day. Since we know from the literature that there are approximately 4.6e6 to 1.4e8 CTLs in Waldeyer's ring directed against the Blast stage (see Supplemental Table 1), we can estimate the average time between kills for these CTLs to be between 60 and 380 years. (It is worth noting here that B-cells in lymph nodes are moving rapidly [34], therefore the number of B-cells that a lytic B-cell could come into contact with may be higher than 20. Given the burst size for a B-cell of ∼10^4^ virions, the number of infected cells could be 10-fold higher; however that does not significantly affect the conclusions here). Under this scenario, therefore, almost the entire CTL population would never see its target again after resolution of the initial acute phase. This is not consistent with published information that ∼10% of CTLs in tonsils against the Blast stage express activation markers [35]. To sustain this response, therefore, requires amplification of the virus beyond those produced by lytic B-cells. We have previously presented biological and modeling data to support the idea that EBV must replicate in epithelial cells to account for the levels of virus shedding we observe in saliva [5]. Figure 7B shows the pie chart where we assume that each bursting B-cell infects epithelial cells, which in turn release virus that infects ∼10,000 B-cells. Here, flow in is dominated by infection and flow out by CTL killing, which is close to 100,000 cells per day. Under this scenario a CTL encounters a target every 46 days to approximately 3.8 years. The lower end of this estimate seems a credible time frame for sustaining the response. It is worth noting that it is well within the capabilities of the 7.7e^8^ macrophages of Waldeyer's ring (this lab and [36]) to deal with a daily death toll of this size. Figure 7C shows the predicted mean time between killings for CTLs specific for Blasts in Waldeyer's ring plotted against a wide range of amplification factors.

We conclude that amplification of EBV, possibly through epithelial infection, is necessary to account for the observed biology of the CTL population.

### Parameters and outcomes

**Figure 8:**
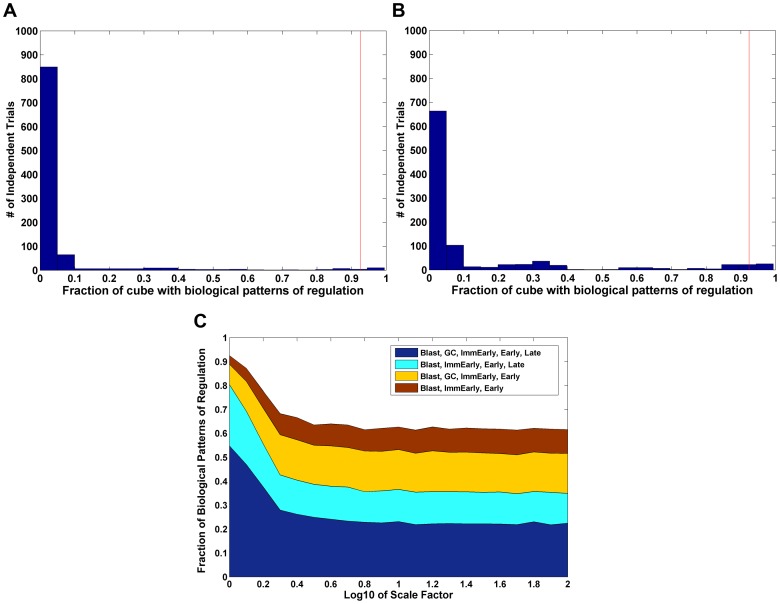
Validation of CPM. A) A permutation test was performed 1000 times where the values for the parameters were randomly scrambled. For details see text. The histogram shows the number of examples where different fractions of biologically plausible patterns of regulation were seen. Note that only 13 scrambled parameter sets had an equal or higher level of biologically plausible patterns of regulation as compared to the biologically plausible parameter set (p-value of 0.013). B) A permutation test was performed 1000 times in which all parameters are permuted (as in A), with the exception of the values for *c_i_*. Note that only 33 scrambled parameter sets (holding *c_i_* constant) had an equal or higher level of biologically plausible patterns of regulation as compared to the unscrambled sets (p-value of 0.033), allowing us to reject the hypothesis that the observed patterns of regulation were determined by the values for net antigenicity. C) The range of values for each parameter was increased by up to 100-fold above what was biologically plausible and the fraction of biologically plausible patterns measured. The fraction of the parameter space which produces plausible patterns quickly falls off, demonstrating that the default set of parameters was very close to optimal.

One concern is that the patterns of regulation described above are somehow intrinsic properties of the model and not dependent on the biological validity of the value ranges we have estimated for the parameters. We have performed several control computational tests to address this. First, we took the values for each parameter and randomly scrambled them between the different stages. For each parameter, there are 6 values, one for each stage. Scrambling these values produces 6! = 720 possible permutations for each parameter. This was performed for each of the four parameters: *r, f, a* and *c*. Each randomized value for a given parameter is then in turn grouped with randomized values for the other three parameters. Since there are in total 4 parameters with 720 permutations each, there are 720^4^ (∼2.7e11) possible permutations of this type. We randomly selected 1000 of these permutations, constructed a parameter cube for each, and sampled each as before. Of the 1000, 13 had a percentage of sampled points with a biologically plausible pattern greater than or equal to that obtained with the unscrambled values (red line in Figure 8A). This gives an empirical p-value of 0.013 and allows us to reject the null hypothesis that randomly chosen parameter estimates would perform as well.

A further concern is that the patterns of regulation we observe are driven by the values we have derived for the net antigenicities, *c_i_*. To address this, we have performed a permutation test in which all parameters, except *c_i_*, are permuted as above. Holding the net antigenicities constant and permuting the other parameters degrades performance over 96% of the time (Figure 8B). This gives an empirical p-value of 0.033 allowing us to reject the hypothesis that the observed patterns of regulation were determined by the values for net antigenicity. We also examined the degradation in performance when only a single parameter is permuted. Since there are six stages, there are 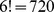, including the permutation which leaves everything in place. The fraction of the time a permutation performed at the same level or better than the unpermutted cube for *r*, *f*, *a* and *c* respectively were 0.149, 0.0875, 0.25 and 0.00278. In summary, although *c* is the most critical parameter, it is not sufficient to account for the observed patterns of regulation.

The ranges of our parameter values were chosen to be as biologically accurate as possible. If this and the model are correct then we should predict that performance will rapidly degrade if we arbitrarily extend the value ranges. To test this, the ranges were increased by up to 100-fold and the fraction of biologically plausible outcomes measured. As may be seen in Figure 8C, the actual set of parameters used was very close to optimal in terms of producing biologically credible patterns; this success quickly falls off within 0.2 logs. This is good evidence for the specificity of the model since it only works optimally at, or close to, a biologically tenable range of values for the parameters and conversely validates the quality of our parameter values.

### How robust is the non-regulation of memory?

Latently infected memory B-cells produce a single viral protein only when undergoing cell division, which takes place perhaps once every 30 days. Furthermore, this protein is produced in low quantities and is poorly presented, if at all [37], [38], [39]. Consequently, these cells are thought to be weakly antigenic. One advantage of a mathematical model is that it can provide quantitative answers to biological questions. In this case, we can ask how weakly antigenic does the memory compartment have to be relative to the other stages in order to produce the biologically expected pattern of regulation?

We took the 10,000 random points in the parameter cube that we sampled for Figure 3 where none showed regulation of the memory stage, and for each of these we computed how much the value for memory net antigenicity *c*_memory_ would have to increase before the stage became regulated. We found that memory remained unregulated if it is at least two orders of magnitude less antigenic than the other stages.

## Discussion

The studies presented here suggest a shift in our understanding of EBV persistence. The mechanism of EBV infection is well understood to involve a cycle of infected stages, but until now it was believed that EBV persists solely because it resides in resting memory B-cells that cannot be recognized by the immune response. Previously, we were able to describe this cycle of infection in terms of a set of differential equations (the cyclic pathogen model or CPM) and show that the solution of these equations at steady state produced one and only one solution that was stable and biologically possible [21]. Put simply, the CPM shows how the rates governing such processes as proliferation, death and differentiation of infected B-cells,, amplification of the virus, and proliferation, loss and killing efficiency of the immune response collectively determine a stable set point for the coexistence of the host and the pathogen. In doing so, it gives us the key to understanding the sizes of infected populations and which fall under CTL regulation. We proposed that the stable set point described by the CPM represented persistent infection. In this paper we have now validated this assertion.

The CPM shifts the focus of persistence from the immunological invisibility of the memory compartment to the cycle of infected stages. The form that persistence takes is a function of the properties of all the infected stages in the cycle and the immune responses to them. Thus the model predicts that for EBV, persistence is possible even with a strongly antigenic infected memory compartment, however the model only works correctly in predicting the whole biologically observed pattern of regulation if the memory compartment has very low antigenicity. So:

1. If you change the antigenicity of the memory compartment so that it is regulated the overall pattern of regulation becomes non-biological. Specifically, we see that the Immediate early stage is frequently not regulated. This is important validation for the model because biologically this stage is always regulated and as such it was assigned a high value for antigenicity in the model. This demonstrates once more that whether or not a stage is regulated is not a function of its assigned antigenicity but rather a consequence of the complex interactions of all the components of the model.
2. You can achieve regulation of the memory compartment even at low antigenicity if you manipulate other parameters, e.g., for the GC, but this requires those parameters to be at biologically untenable values (not shown).

Thus, the biologically correct low antigenicity of the memory compartment and the correct pattern of regulation are integrally linked. The model says you can't have one without the other.

It is important to stress that our analysis to date does not address how acute infection resolves into persistence, nor the role of stochasticity in these dynamics (see Box 4). In this paper we have studied the application of the CPM to the equilibrium state of normal chronic EBV infection and showed that it is capable of recapitulating important structural features of this infection. The CPM contains simplifications that make it mathematically tractable. Perhaps the most prominent of these is the simplification of CTL dynamics and the omission of humoral and innate immune response. In addition, the parameters of this model (*r_i_*, *f_i_*, *a_i_*, *c_i_* and *b*) likely exhibit different values in the acute and chronic phases. Thus, while preliminary studies of the dynamics of the CPM show broad correspondence to our limited knowledge of acute EBV dynamics, detailed validation of a model of acute EBV dynamics is outside the scope of the current study. To highlight these issues we have given them a more detailed treatment in Box 4 where we have provided details on the simplifications and limitations of the model and some relevant preliminary studies. Given the issues listed in Box 4 one may ask the question “how can we be sure that the mathematics of CPM is the ‘correct’ model of EBV?”. However, this is the wrong question. Quoting the famous words of George Box, “Essentially, all models are wrong, but some are useful” [40]. The better question to ask therefore is, “is the CPM model of EBV persistence useful?”. The evidence presented in this paper shows that the model is very useful. Although it has 25 parameters and 64 possible patterns of regulation, the top four patterns predicted were the 4 biologically credible ones. Furthermore, the ranking of the four agreed exactly with what is observed biologically and accounted for essentially all of the parameter sets (93.1%). Non-biological patterns were effectively not seen. Given that no parameter(s) forces this outcome, the chances of obtaining this result with an “incorrect” model are vanishingly small. Additionally, the model accurately predicted the actual sizes of the unregulated memory and GC compartments, a striking result given that the actual range in sizes of the six populations covers more than 4 logs (see Figure 1B). Lastly, when parameter values were scrambled or manipulated so they were outside the biological range or even when a non-biological value was assigned to the antigenicity of a single population (i.e., intentionally making the model “incorrect”), the outcomes rapidly and dramatically became non-biological. Taken together, these results reinforce the conclusion that it is not credible that we could have fallen upon this result as a fortuitous outcome of an “incorrect” model. The final test of the utility for a model is if it provides significant new insights and CPM indeed achieves this in providing a new and completely biologically consistent explanation for persistence, i.e., its dependence on the cycle of infection. The main conclusion of our mathematical analysis is the understanding that if a pathogen has a cycle of infection that is regulated by the immune response, it then has the possibility to use this cycle to establish an extremely stable persistent infection. As such, EBV might be unique because it relies on the biology of the B-cell which provides the platform for the cyclic behavior, i.e., the virus is simply exploiting the normal cycle of B-cell activation, memory, reactivation and differentiation. This is not to say that a cycle of infection is the only way for a pathogen to persist, nor even that it's a better way, simply that it is an evolutionary niche that EBV has occupied because of the nature of the cell type it infects - the B lymphocyte. The mathematics then tells us that this is sufficient to sustain a stable persistent infection, i.e., a cycle of infection is sufficient but by no means essential for persistence. It will be interesting to see if other pathogens are able to exploit this behavior or if it is unique to EBV.

**Box 4. Simplifications and Limitations of the Model**

There are three major areas of simplification in the equations of the CPM. These involve the description of the T-cell response, application to acute infection (dynamics) and the consequences of stochasticity.

1. The T-cell response.
  The CPM uses a simplified term for activation of T-cells by antigen: 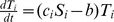, i.e., the proliferation rate of each CTL population is simply proportional to the size of its cognate infected population and the amount of antigen, and therefore not saturable.
  We have been able to extend the theoretical conclusions of this model in a mathematical analysis where we consider a very general form of this equation 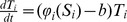. This is referred to as the non-linear cyclic pathogen model. It allows the proliferation rate to be any increasing function of antigen, including the case where the response becomes saturated. We find that CPM can be extended to these very general classes of dose-response curve for CTL activation that more closely resemble biology. Specifically, for any parameter values, there is still a unique stable fixed point, with the exception that under sufficient immune suppression there is the possibility of cancer-like runaway growth. Thus, the conclusions of this paper remain robust in the face of added complexity to the model. This work is detailed in [52]. However, to apply this more general model to the specifics of EBV would require data characterizing the functions which correspond to *in vivo* proliferation rates for the different CTL populations. To the best of our knowledge, such data are not available at this time. As to the specifics of which stages are regulated, there is no way to derive the shapes of the functions in the non-linear case from currently available data except by saying that at the equilibrium point that we observe they work out to have the same net antigenicities for the regulated stages, thus the same pattern of regulation is observed.
  We have also treated each stage as having a single, unique CTL response. In reality stages have CTL responses to multiple antigens and shared antigens across stages. Modeling this sort of “antigenic cross-talk” will require insights into new systems of differential equations.
  The CPM also encapsulates the immune response to each stage into a single CTL population. The real T-cell response is considerably more complex. Furthermore, we only consider the CD8/CTL response; we do not take CD4 T-cells into account, nor do we include a humoral or innate response. We also do not include discrete CTL sub-populations such as effector, central memory and effector memory, all of which have different life-spans and activation requirements [49], [50], [51], or the role of APCs. The biological relationship between these compartments is not understood in detail at this time and modeling these factors will require a significant increase in biological understanding and model complexity.
  Nevertheless, despite these caveats the CPM accurately predicts biologically accurate regulation profiles >90% of the time when credible parameter values are used, accurately predicts infected population sizes and degrades rapidly when parameter values become non-biologic. This suggests that our simplifications of CTL activity have encapsulated sufficient of the properties of CTL to produce useful outputs.
2. Acute dynamics.
  In this paper, we have limited our study of CPM to defining the steady state of persistent chronic infection; we have not used the mathematical model to address the dynamics of acute infection. The primary reason for this is the dearth of biological information for informing parameter estimates. Even more crucial is that we have no data on the course of infection over the first ∼5 weeks. Clinical/biological studies are only initiated when patients become sick and we have shown that at this point the infection is resolving [53]. It is unknown if there is a single peak of infection or multiple oscillations before resolution. We do know that once the infection begins to resolve it does not show large oscillation in the level of infection but takes at least one year to reach a steady state [53].
  We have performed some preliminary simulations to look at acute dynamics and find that the CPM appears sound resolving acute infection with dynamics that broadly resemble what is known biologically. Using biologically credible parameters, the model proceeds from initial infection to the stable fixed point in approximately 1–2 years without showing large, continuing oscillations (preliminary observations, not shown).
  In the CPM, the biological processes are modeled using the constant coefficients *r_i_*, *f_i_*, *a_i_*, *c_i_* and *p_i_*, and we expect that some of these processes will have different rates in the acute phase and may even vary throughout acute infection. For example, *f*_memory_ is probably higher in the acute phase. In contrast, chronic EBV persistence appears to be a system in equilibrium, allowing us to treat these rates as constant. As a consequence, we consider the present work to be validation of the model only for the chronic phase and expect that accurate dynamical modeling will require further elaboration of the model.
3. Stochasticity in acute infection.
  For the same reasons discussed in 2) we believe it is premature to study the effects of stochasticity in the acute phase as we do not know how these play out *in vivo*, i.e., whether fluctuations in parameter values can lead to extinction during acute infection. Specifically, it is unknown whether exposure to EBV consistently leads to persistent infection or fails at some rate, i.e., multiple infection events are required for successful establishment. Thus,we do not know what the expected outcome is for the model. Preliminary simulations show broad concordance with the limited data available (see above).
Stochasticity in persistent infection.
  We know that once persistent infection is established it is extremely stable and lasts for life, i.e., there are no naturally occurring perturbations that drive it to extinction. Biologically, it is possible that stochastic fluctuations could result in the temporary ablation of one or more infected stages. This is likely for the Early lytic and Late lytic stages since these have extremely low numbers of cells at equilibrium (see Figure 1B). However, we can ask why ablation of one stage does not propagate to the next and thence to the entire cycle. This reflects the ability of all the infected stages to act as a reservoir for the other stages thus assuring persistence, i.e., the cycle of infection is the cause of persistence.
  We have not undertaken extensive studies of the results of introducing stochastic effects into this model. Preliminary studies show that the fixed point is robust with respect to stochastic effects with perturbations largely damping out over the course of 3–6 months (data not shown).

In sum, we believe the fact that CPM works well as a model of chronic EBV infection validates the use of these simplifying assumptions (see the discussion for a detailed consideration of this issue). Each of these simplifications presents a possible direction for future elaboration of the model. However, any addition of further complexity will have to be done in such a way that the model performs at least as well, if not better, than CPM.

The persistence engendered by the cycle of infection provides the possibility for lifelong infection with the potential for continuous horizontal spread. However, this stability also confers another crucial advantage, namely it allows the pathogen to persist at extremely low levels where stochastic variation, that might occasionally drive a single infected population to extinction, can be tolerated. Thus for an acutely infecting virus, once the immune system clears the infected target population, the infection stops. However, in the case of a cyclic behavior, if any infected stage is temporality cleared, it can be repopulated by the cycle. This ability allows EBV to persist at extremely low levels (<1 infected cells/250 ml of blood in some individuals [41]), thus minimizing any deleterious impact on the host on whose survival the virus depends for lifelong persistence. The result is that EBV is a highly, if not the most, successful human pathogen infecting >95% of the human race for life. It is important to stress that the stability we demonstrate is not an assumption of the model, nor do we need to “fine tune” our parameter set to achieve it; stability is a purely emergent property. Thus, the GC model is not only experimentally validated, but also mathematically consistent, and therefore sufficient to produce stable persistent infection.

### Predictions and new biological insights

For the first time we have a detailed, mathematically consistent model of EBV persistence. The advantage of such a model is that it now allows us to make quantitatively precise predictions about infection, i.e., observe the extent to which the mathematics constrains the biological possibilities. In the results section we have presented such arguments with respect to the size and antigenicity of the memory compartment, whether or not infected GC B-cells come under immune regulation and the possible role of viral amplification in epithelium between the Late lytic and Blast stages. From a practical stand point the model can also be used to make predictions about interventions and how effective they must be to alleviate or prevent EBV infection and associated diseases, and what their probability of success might be. Generally speaking, CPM predicts that it will be extremely difficult to clear EBV infection once established. This is because it is the complete cycle of infection, not any one stage, which is important for persistence. Consequently, any treatment regimen must reduce the value of *R*_0_ (the net amplification achieved by one circuit of the cycle) to less than 1. The value of *R*_0_ depends critically on the amplification factor at the Late lytic stage which is likely to lie in the range ∼10^4^–10^6^. This means that persistent infection is not only robust with respect to random variations in populations as we have discussed, it is also robust with respect to large changes in the parameter values, e.g., those induced by the administration of an anti-viral or vaccine. In order to eliminate EBV infection, such treatment would have to reduce viral production by a factor of 10^4^ or greater. This will be a difficult task given the complicated PK/PD issues involved in administering antivirals and the inability of anti-herpesviral drugs to dramatically reduce EBV production for a sustained period of time [42], [43].

One consequence of CPM that may not be self-evident to a biologist is that if an infected stage is being regulated by a CTL response, then the level of that stage is controlled solely by its antigenicity and the decay rate of the CTL response - and nothing else (see also [44]). There are several consequences of this. For example:

1. Burkitt's lymphoma and Hodgkin's disease are believed to descend from latently infected GC B-cells which have failed to successfully differentiate into a resting memory state [2]. If correct, then decreasing the infected GC population should reduce the risk of these diseases. The CPM predicts that if the GC stage is under CTL regulation, then there are two ways to decrease the number of infected GC B-cells. The first is to increase their net antigenicity, i.e., the ability to stimulate a CTL response, and the second by reducing the production and/or dwell time of the infected GC B-cells. However, an important and again not intuitive insight from CPM is that the later will only take effect when the supply of infected GC B-cells is reduced below that which is required to stimulate a T-cell response. As a consequence, we can estimate from CPM that any reduction in the level of infected GC B-cells requires a 3-fold steeper decrease in production/dwell time, e.g., a 2-fold decrease in infected GC B-cells requires a 6-fold reduction in production/dwell time.
2. We have observed that the level of infected GC B-cells in tonsils from patients with malaria is increased 50-fold over that in normal tonsils (unpublished observations). Two established consequences of malarial infection are that it can activate B-cells [45] and is immunosuppressive for T-cells [46]. While one might assume a priori that either of these phenomena could affect the level of infected GC B-cells, the CPM states unequivocally that the increase in the GC population is due solely to the immunosuppression of T-cells; that is, there is a lower net effective antigenicity of GC B-cells in the presence of malaria. Furthermore, if this is a general immunosuppression, CPM predicts that there will also be a 50-fold increase in all the regulated stages, Blast, Immediate early, Early and possibly Late lytic.

A generalization of our analysis is that if a pathogen can establish a cycle of infection, it has the potential to become persistent. This can be true of any virus. For example, an acutely infecting virus like influenza can be thought of as having a very simple cycle, i.e., infectious virus cycling through infected cells and back to virus. Why doesn't influenza establish a persistent infection? The answer is twofold. First, when potent neutralizing antibodies arise they effectively break the cycle of infection by removing all infectious virus. This break is permanent because the antibody response persists long after the antigen is cleared. Second, the virus does not have multiple infected stages that can re-establish the cycle when it is interrupted by the immune response. In the case of EBV, potent neutralizing antibodies arise but are apparently unable to effectively clear all of the virus. We know this because despite the presence of potent neutralizing antibody in the serum, infectious virus can readily be isolated from the saliva, and newly infected naive B-cells are routinely present in the tonsils of healthy carriers of the virus. We have attributed EBV's ability to establish persistent infection to the existence of a cycle of infective stages. We can in turn attribute the existence of that cycle to the failure of antibody to provide a sterilizing response.

Why it is not possible to produce a sterilizing level of neutralizing antibody to EBV is unclear, but is crucial in allowing the cycle of infection to proceed. In our model we have encapsulated the steps between lytic infection and blasts because this is an area where we are still lacking detailed information about the intervening steps. We do not know if the virus effectively avoids neutralization because of compartmentalization of infection and serum antibodies, or because virus transmission is through cell-to-cell contact, for example. As we have argued above and previously [5], it seems inevitable that infectious virus is amplified in the epithelium on the way to infecting new naive B-cells. A complicating but very interesting issue that arises is the possible role of epithelial cells in abrogating the sterilizing effect of neutralizing antibody. It is known that antibody that neutralizes B-cell infection actually favors epithelial cell infection [47], thus giving a positive feedback loop in response to neutralizing antibody (for modeling of this effect see [24]).

It seems, therefore, that modeling has produced several compelling reasons to believe that epithelial infection plays a central role in persistence. It will be important to better understand the exact relationship between the route(s) taken by infectious virus between B-cells and epithelium, since it relates directly to the pathways by which EBV must enter the tonsil lymphoepithelium during initial acute infection. The level of infected memory B-cells in the peripheral blood of healthy carriers is stable ([5], [6] and unpublished observations). There is no detectable decline (or expansion). By comparison levels of shed virus in saliva are extremely variable (by up to 4 logs) [5]. If the levels in saliva truly reflected infectious virus for naive B-cells, then we would expect to see a large variation in the ratio of infected Blasts to GC to memory B-cells, with the variation in the level of infection dampening down as the virus traverses the infected stages into memory. However, we see no evidence of this in our analysis of a large numbers of tonsil samples [41]. This suggests that the rate of new infection is relatively constant and independent of the wild fluctuations of virus shedding in saliva.

The validation that we have offered in this paper makes a strong case that the CPM is able to capture the gross features of the architecture of persistent EBV infection, and gives a first principled quantitative explanation of how this architecture produces persistent infection. It will be important now to extend this work to the dynamics of acute infection and more detailed description of the biology, particularly of the immune response to EBV.

## Methods

### Parameter values

For CPM, we assume that all of the significant biology for EBV occurs in the lymphoid tissue of Waldeyer's ring. Therefore all CPM parameter values are for the entire Waldeyer's ring. We have not attempted to include the peripheral blood, which contains relatively few infected cells, nor the peripheral lymphoid tissue where the level of infection is markedly lower [41]. We assume, but have not tested, that the dynamics of infection in Waldeyer's ring are representative of the whole body. We have also omitted the naive B-cells that the virus infects because, based on our own calculations on the number of naive B-cells in Waldeyer's ring (∼5e9, see Supplemental Table S1), the supply of new naive target cells is not a limiting factor. That is, EBV infects at most ∼1e6 naive B-cells out of a total of ∼5e9, and given the fact that immature B-cells can replenish and effectively buffer the naive compartment [48], we do not expect such a relatively small amount of new infection to significantly reduce naive B-cell numbers.

We only consider the CD8/CTL response; we do not take CD4 T-cells into account, nor do we include a humoral or innate response. Furthermore, we do not include discrete CTL sub-populations such as effector, central memory and effector memory, all of which have different life-spans and activation requirements [49], [50], [51]. We also do not include any model of CTL exhaustion after chronic stimulation nor of EBV-induced inhibition of antigen processing/presentation, i.e., the known decrease in presentation of lytic antigens from Immediate early>Early>>Late [33]. This effect is encapsulated into net antigenicity.

Traditionally, there are two methods by which the values for the parameters in a mathematical model may be determined. In the first method, one searches for values that give an outcome for the model that most closely approximates what is seen biologically. In essence this is empirical fitting. The second method attempts to directly or indirectly measure the individual parameters experimentally. This typically produces a range of observed values due to technical limitations and variation that is a natural property of the biological system itself for any given human population. In this work we have followed the second approach. We have used our own laboratory's work together with an extensive search of the currently available literature for experiments that either directly measure the parameters we are interested in or allow for an indirect calculation. A complete list of parameter values is given in Supplementary Table S1. This includes discussion of their origin (together with references) and any potential limitations.

The parameter *r*_late lytic_ encapsulates all of the processes that go on between the burst of a Late lytic cell and the production of new naive Blasts. This includes free virus, the role played by infected epithelium, and the humoral response against free virions. Analysis of this parameter, its size and the implications for viral replication are considered in the results section.

The parameter *c_i_* or “net antigenicity” encapsulates an overall efficiency of promoting stage-specific CTL activation and proliferation. Because it encapsulates so many biological processes, it is difficult to obtain a single laboratory measurement for it. However, as the model makes clear, the size of a regulated stage is determined by net antigenicity together with the death rate for CTLs; a regulated stage stabilizes at exactly the level where the antigenic population provokes just enough activation and proliferation of CTL to balance losses. Consequently, for regulated stages, we have derived a value for “net antigenicity” from the size of the infected population and our estimate of the CTL decay rate. It is worth noting that the ranking of these derived values for the lytic stages agrees with the published ranking based on avidity of peptide binding [33].

### The parameter space/cube

To investigate if the model exhibits the observed regulation patterns when biologically credible values are assigned to the parameters, we developed a parameter cube as follows. The parameters of the CPM are listed in Box 2. Parameters *r_i_*, *f_i_*, *a_i_*, *c_i_* and *p_i_* have stage-specific values, that is, the model requires a value for each of these at each of the six stages. The parameter *b* describes the decay rate for a CTL population in the absence of antigen which we assume is not stage specific and therefore has a single value which is applied to all stages. Thus, the model has 31 distinct parameters. At equilibrium the value of the parameter *p_i_* only affects the size of the CTL populations, not the infected populations (see Box 3). Therefore, the pattern of regulation is determined by the 25 remaining parameters *r_i_*, *f_i_*, *a_i_*, *c_i_* and *b*.

We are able to assign precise values for 8 of the 25 parameters. *r_i_* gives the gain in proceeding from one stage to the next and thus is equal to 1 for all stages except Late lytic. *a_i_* gives the difference between the death and proliferation rates at stage *i*. Since the memory B-cell compartment is stable, we assume *a*_Memory_ = 0, i.e., the birth and death rate are equal. Further, the net antigenicity of the memory compartment is vanishingly low. Because of this, there was no need to work with a range of values for this parameter. We have therefore assigned a single value which we justify below. We have also assigned *a*_late lytic_ = 0 under the assumption that once initiated, the Late lytic process always ends in death. This leaves 17 parameters for which we cannot determine the exact values. We have defined the ranges of these parameters using values from the literature and unpublished results from this laboratory (see Supplementary Table S1). Collectively, these fixed values and defined ranges define our concept of biologically credible values. We are thus concerned with 24 stage-specific parameters, 8 of which are assigned fixed values and 16 of which are allowed to vary over fixed ranges, plus *b*. This set of values can be thought of as a 25-dimensional space; we refer to this as the *parameter cube*. Choosing a biologically credible value for each of the parameters is the same thing as choosing a single point in this parameter cube. We are thus able to probe the behavior of the model when confronted with biologically credible parameter values by choosing random points from this cube and running the model using each of these randomly chosen points.

## Supporting Information

Table S1 **CPM parameters.** This table lists and discusses relevant biological and model parameters used in the CPM. Experimental references are cited where applicable.
(DOC)
