# Supplementary material for: The Cycle of EBV Infection Explains Persistence, the Sizes of the Infected Cell Populations and Which Come under CTL Regulation
Source: PLoS Pathog. 2013 Oct 17;9(10):e1003685. doi: 10.1371/journal.ppat.1003685 (PMC3798424; doi:10.1371/journal.ppat.1003685)
Supplement: Table S1 — CPM parameters. This table lists and discusses relevant biological and model parameters used in the CPM. Experimental references are cited where applicable. (DOC) [file ppat.1003685.s001.doc]

**Supplementary Table S1. CPM Parameters.**

| **Stage** | **Parameter** | **Symbol** | **Valuea** | | | **Classb** | **Notes** |
| --- | --- | --- | --- | --- | --- | --- | --- |
|  |  |  | *min* | *pref.* | *max* |  |  |
|  |  |  |  |  |  |  |  |
| B-cells |  |  |  |  |  |  |  |
|  | B-cells in Waldeyer’s Ring (WR) |  |  | 1e10 |  | est. | The total number of mature B-cells in WR has been estimated based on our own measurements. |
|  | % naive out of total B-cells |  |  | 50 |  | exp. | Tonsil B-cell sub-populations represent average measurements routinely observed in our lab. |
|  | Naive B-cells in WR |  |  | 5e9 |  | est. | The total number of each B-cell subset is based on the experimentally observed subset distribution and the previous determination of total B-cells in WR. |
|  | % GC out of total B-cells |  |  | 24 |  | exp. |  |
|  | GC B-cells in WR |  |  | 2.4e9 |  | est. |  |
|  | GC B-cells per GC |  | 0.5e5 | 1e5 | 2.5e5 | exp. |  |
|  | % memory out of total B-cells |  |  | 24 |  | exp. |  |
|  | Memory B-cells in WR |  |  | 2.4e9 |  | est. |  |
|  | % plasma out of total B-cells |  |  | 2 |  | exp. |  |
|  | Plasma B-cells in WR |  |  | 2e8 |  | est. |  |
|  |  |  |  |  |  |  |  |
| EBV-infected Blast |  |  |  |  |  |  |  |
|  | Infected Blasts per naive B-cell |  | 1e-7 | 25e-7 | 300e-7 | exp. | The ranges of EBV-infected cells in each B-cell subset are consistent with measurements routinely observed in our lab. The preferred value represents our view of a representative “average” value. |
|  | amplification factor | r_Blast | 1 |  | 1 | exp. | This parameter controls the amplification of infection after differentiation into the next stage. EBV is replicated during the lytic stages and released after the Late stage. Thus, all other stages have an amplification factor of 1. |
|  | rate of flow to GC per day | f_Blast | 0.5 | 1 | 2 | est. | This parameter dictates the rate of flow into the GC compartment. The minimum value represents 2 days spent in the blast state, while the maximum value is 12 hours. |
|  | net proliferation / death rate | a_Blast | -2 | -1 | 0 | est. | This parameter encapsulates both the net proliferation (negative values) and death (positive values) of infected Blasts. Cells range from not dividing (0), to dividing 2/day (-2). |
|  |  |  |  |  |  |  |  |
| EBV-infected GC B-cell |  |  |  |  |  |  |  |
|  | infected GC B-cell per GC B-cells |  | 1e-7 | 100e-7 | 3000e-7 | exp. |  |
|  | amplification factor | r_GC | 1 |  | 1 | exp. |  |
|  | rate of flow to memory per day | f_GC | 1 |  | 4 | est. | This parameter dictates the rate of flow into the memory compartment. These values represent a range of 6 to 24 hours spent in the GC, before entering the memory compartment. |
|  | net division | a_GC | -2 | -1 | 0 | est. | This parameter encapsulates both the net proliferation (negative values) and death (positive values) of infected GC B-cells. Infected GC B-cells are assumed to not die in the GC, thus this value represents rate of proliferation. Cells range from not dividing (0), to dividing 2/day (-2), based on the observed GC B-cell cell cycle time of a minimum of 12 hours . |
|  |  |  |  |  |  |  |  |
| EBV-infected Memory B-cell |  |  |  |  |  |  |  |
|  | infected memory B-cell per memory B-cells |  | 1e-7 | 100e-7 | 3000e-7 | exp. |  |
|  | amplification factor | r_Memory | 1 |  | 1 | exp. |  |
|  | rate of flow to Immediate early per day | f_Memory | 0.01 | 0.03 | 0.1 | est. | This parameter dictates the rate of flow into the Immediate early compartment. At the high end, this range argues for ~100 days spent in the memory compartment, while the low end suggests quick differentiation into IE. The preferred value represents ~30-40 days, which is comparable to the experimentally observed rate of homeostatic division of normal uninfected memory B-cells . |
|  | net division and death | a_Memory | 0 |  | 0 | exp. | This parameter encapsulates both the net proliferation (negative values) and death (positive values) of infected memory B-cells. Infected memory B-cells are assumed to follow normal B-cell homeostasis, with a balance between proliferation and death. |
|  |  |  |  |  |  |  |  |
| EBV-infected Lytic B-cell: Immediate early |  |  |  |  |  |  |  |
|  | infected plasma per memory B-cells |  | 1e-7 | 100e-7 | 3000e-7 | est. | The frequency of EBV-infection in the plasma compartment is estimated to be comparable to that of the GC and memory compartments, based off of qualitative analysis of limiting dilution PCR. |
|  | % of EBV-infected plasma cells expressing Immediate early proteins |  | 10 | 15 | 25 | exp. | These measurements represent data from two human tonsils. |
|  | total Immediate early at steady-state |  | 50 | 250 | 500 | est. | The total number of EBV-infected cells in Immediate early at equilibrium was extrapolating by the range of infection frequency. The final range was then restricted to plausible biological values. |
|  | amplification factor | r_ImmE | 1 |  | 1 | exp. |  |
|  | rate of flow to Early per day | f_ImmE | 2/3 | 1 | 2 | est. ­ | This parameter dictates the rate of flow into the Early compartment. The minimum value represents 36 hours spent in the Immediate early state, while the maximum value is 12 hours. |
|  | net division | a_ImmE | 0.1 |  | 0.5 | est. | Since the Immediate early state does not proliferate, this parameter controls the death rate. It is unclear if EBV is subject an abortive lytic cycle. The chosen range has 10% cell death at the low end, and 50% at the high end. This was estimated from in vitro experiments that looked at total death from Immediate early -> Late. Based on our experimentation, these values have little to no effect on the observed regulation profiles. |
|  |  |  |  |  |  |  |  |
| EBV-infected Lytic B-cell: Early (E) |  |  |  |  |  |  |  |
|  | % of EBV-infected plasma cells expressing Early proteins |  | 1 | 5 | 12 | exp. | These measurements represent data from two human tonsils. The minimum value is approximate since it is based on detecting a single positive signal in a limited dilution analysis. |
|  | total Early at steady-state |  | 5 |  | 50 | est. | The total number of EBV-infected cells in Early at equilibrium was derived by first extrapolating the range from the infection frequency, and then restricting the values to a plausible biological range. |
|  | amplification factor | r_Early | 1 |  | 1 | exp. |  |
|  | rate of flow to Late per day | f_Early | 1 | 2 | 4 | est. | This parameter dictates the rate of flow into the Late compartment. The minimum value represents 24 hours spent in the Immediate early state, while the maximum value is 6 hours. |
|  | net division | a_Early | 0.1 |  | 0.5 | est. | As mentioned above for a_Immdiate early, this value was estimated from in vitro measurements of total cell death from Immediate early -> Late. This range. It is not clear if EBV has an abortive lytic cycle, however these values have little to no effect on the observed regulation profiles. |
|  |  |  |  |  |  |  |  |
| EBV-infected Lytic B-cell: Late (L) |  |  |  |  |  |  |  |
|  | % of EBV-infected plasma cells expressing Late proteins |  | 1 | 1 | <3 | exp. | These measurements represent data from two human tonsils. The minimum value is approximate since it is based on detecting a single positive signal in a limited dilution analysis. |
|  | total Late at steady-state |  | 2 | 5 | 10 | est. | The total number of EBV-infected cells in Late at equilibrium was derived by first extrapolating the range from the infection frequency, and then restricting the values to a plausible biological range. |
|  | amplification factor | r_Late | 10 | 1e5 | 1e6 | est. | The amplification factor encapsulates viral production and release of virus by the bursting Late lytic infected cell, as well as the potential subsequent infection, production and release of virions by the epithelium (see Discussion). Note that this amplification factor speaks to the total number of productive infection events, which is assumed to be significantly lower than total infection virions released (as not every virion will infect a naive B-cell). The min. value assumes that the Late lytic burst only infects surrounding naive B-cells, with the high end argues for significant amplification by the epithelium. |
|  | rate of burst (viral release) per day | f_Late | 1 | 2 | 4 | est. | This parameter dictates the rate of Late bursts. The minimum value represents 24 hours spent in the Late state, while the maximum value is 6 hours. |
|  | net division | a_Late | 0 |  | 0 | est. | We assume that once at the Late stage, all infected cells will burst and release virions. Therefore, there is no death of Late B-cells. |
|  |  |  |  |  |  |  |  |
| T-cells |  |  |  |  |  |  |  |
|  | ratio of T-cells to B-cells in WR |  |  | 0.46 |  | exp. | This ratio was taken from published reports and is consistent with measurements routinely observed by our lab. |
|  | T-cells in WR |  |  | 0.46e10 |  | est. | This is estimated based on the previous determination of total B-cells in WR. |
|  |  |  |  |  |  |  |  |
| CTL |  |  |  |  |  |  |  |
|  | CTLs per T-cell in WR |  | 0.1 | 0.2 | 0.3 | exp. | These values are based on CD8/CD3 ratio from human tonsils, which lie close to the preferred value. The min and max represent potential individual variation. |
|  | lifespan in the absence of antigen (b) |  |  | 0.07 |  | exp. (reviewed in ) | This parameter controls the lifespan of a CTL in absence of antigen. One limitation of our model is that we do not differentiate between effector, central memory or effector memory. CPM is simplified such that all CTLs survive for a short time (~14 days) in the absence of antigen, to reflect the dependence of the immune response on the availability of antigen. This value is based on the contraction of effectors after the resolution of an immune response. |
|  | CTLs specific for EBV-infected Blasts (% total CTLs) |  | 1 | 3 | 10 | exp. | This range is based on tetramer staining of CD8 T-cells in human tonsils from patients with chronic EBV. |
|  | CTLs specific for EBV-infected GC B-cells (% total CTLs) |  | 0 | 1 | 5 | exp. | While also based on tetramer staining, it is not clear if CTLs are able to enter the GC to actively kill infected GC B-cells. |
|  | CTLs specific for EBV-infected memory (% total CTLs) |  | 0 |  | 0 | exp. | Aside from brief expression of EBNA1 during homeostatic division, EBV-infected memory B-cells do not express EBV proteins and are not predated by CTLs . |
|  | CTLs specific for EBV-infected Immediate early (% total CTLs) |  | 1 | 5 | 10 | exp. | This range is based on tetramer staining of CD8 T-cells in human tonsils from patients with chronic EBV. |
|  | CTLs specific for EBV-infected Early (% total CTLs) |  | 0.5 | 1 | 2 | exp. | This range is based on tetramer staining of CD8 T-cells in human tonsils from patients with chronic EBV. |
|  | CTLs specific for EBV-infected Late (% total CTLs) |  | 0 | 0.01 | 0.1 | est. | While CTLs against Late lytic cells are rare, they are found in some patients. |

a Note on Value: Ranges are bounded by minimum (min.) and maximum (max.) values, either taken directly from the literature or based on our own estimates. Preferred (pref.) values represent the most likely physiological values, and are not necessarily the experimental mean or the midpoint between min. and max.

b Note on Class: Experimental (exp.) parameters are derived from personal/published measurements and are assumed to be accurate. Estimated (est.) parameters, while also taken from our own experiments and the literature, are approximated with less certainty.

**REFERENCES**

1. Roughan JE, Torgbor C, Thorley-Lawson DA (2010) Germinal center B cells latently infected with Epstein-Barr virus proliferate extensively but do not increase in number. J Virol 84: 1158-1168.

2. Laichalk LL, Thorley-Lawson DA (2005) Terminal differentiation into plasma cells initiates the replicative cycle of Epstein-Barr virus in vivo. J Virol 79: 1296-1307.

3. Laichalk LL, Hochberg D, Babcock GJ, Freeman RB, Thorley-Lawson DA (2002) The dispersal of mucosal memory B cells: evidence from persistent EBV infection. Immunity 16: 745-754.

4. Thorley-Lawson DA, Strominger JL (1978) Reversible inhibition by phosphonoacetic acid of human B lymphocyte transformation by Epstein-Barr virus. Virology 86: 423-431.

5. Allen CD, Okada T, Tang HL, Cyster JG (2007) Imaging of germinal center selection events during affinity maturation. Science 315: 528-531.

6. Macallan DC, Wallace DL, Zhang Y, Ghattas H, Asquith B, et al. (2005) B-cell kinetics in humans: rapid turnover of peripheral blood memory cells. Blood 105: 3633-3640.

7. Wirths S, Lanzavecchia A (2005) ABCB1 transporter discriminates human resting naive B cells from cycling transitional and memory B cells. Eur J Immunol 35: 3433-3441.

8. Hadinoto V, Shapiro M, Greenough TC, Sullivan JL, Luzuriaga K, et al. (2008) On the dynamics of acute EBV infection and the pathogenesis of infectious mononucleosis. Blood 111: 1420-1427.

9. Souza TA, Stollar BD, Sullivan JL, Luzuriaga K, Thorley-Lawson DA (2005) Peripheral B cells latently infected with Epstein-Barr virus display molecular hallmarks of classical antigen-selected memory B cells. Proc Natl Acad Sci U S A 102: 18093-18098.

10. Souza TA, Stollar BD, Sullivan JL, Luzuriaga K, Thorley-Lawson DA (2007) Influence of EBV on the peripheral blood memory B cell compartment. J Immunol 179: 3153-3160.

11. Amon W, Binne UK, Bryant H, Jenkins PJ, Karstegl CE, et al. (2004) Lytic cycle gene regulation of Epstein-Barr virus. J Virol 78: 13460-13469.

12. Ressing ME, Keating SE, van Leeuwen D, Koppers-Lalic D, Pappworth IY, et al. (2005) Impaired transporter associated with antigen processing-dependent peptide transport during productive EBV infection. J Immunol 174: 6829-6838.

13. Al Tabaa Y, Tuaillon E, Bollore K, Foulongne V, Petitjean G, et al. (2009) Functional Epstein-Barr virus reservoir in plasma cells derived from infected peripheral blood memory B cells. Blood 113: 604-611.

14. Bergler W, Adam S, Gross HJ, Hormann K, Schwartz-Albiez R (1999) Age-dependent altered proportions in subpopulations of tonsillar lymphocytes. Clin Exp Immunol 116: 9-18.

15. Boyaka PN, Wright PF, Marinaro M, Kiyono H, Johnson JE, et al. (2000) Human nasopharyngeal-associated lymphoreticular tissues. Functional analysis of subepithelial and intraepithelial B and T cells from adenoids and tonsils. Am J Pathol 157: 2023-2035.

16. Ahmed R, Akondy RS (2011) Insights into human CD8(+) T-cell memory using the yellow fever and smallpox vaccines. Immunol Cell Biol 89: 340-345.

17. Hislop AD, Kuo M, Drake-Lee AB, Akbar AN, Bergler W, et al. (2005) Tonsillar homing of Epstein-Barr virus-specific CD8+ T cells and the virus-host balance. J Clin Invest 115: 2546-2555.

18. Quigley MF, Gonzalez VD, Granath A, Andersson J, Sandberg JK (2007) CXCR5+ CCR7- CD8 T cells are early effector memory cells that infiltrate tonsil B cell follicles. Eur J Immunol 37: 3352-3362.

19. Pudney VA, Leese AM, Rickinson AB, Hislop AD (2005) CD8+ immunodominance among Epstein-Barr virus lytic cycle antigens directly reflects the efficiency of antigen presentation in lytically infected cells. J Exp Med 201: 349-360.
